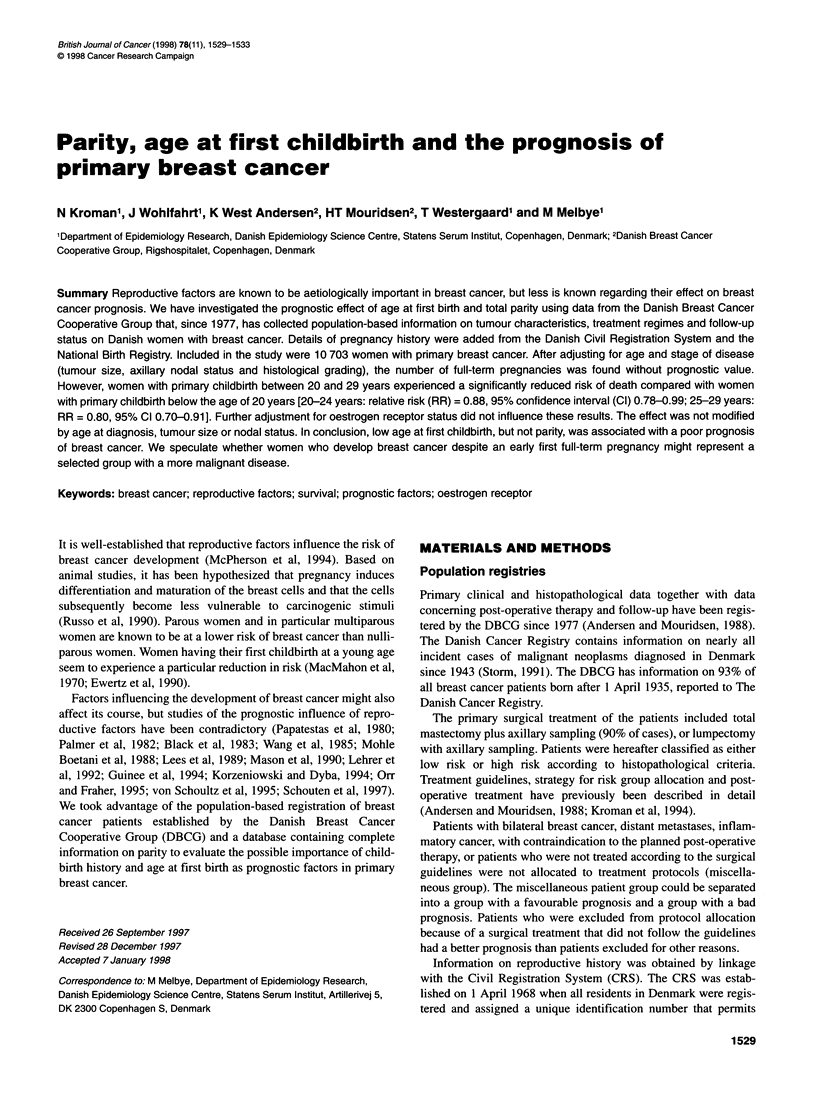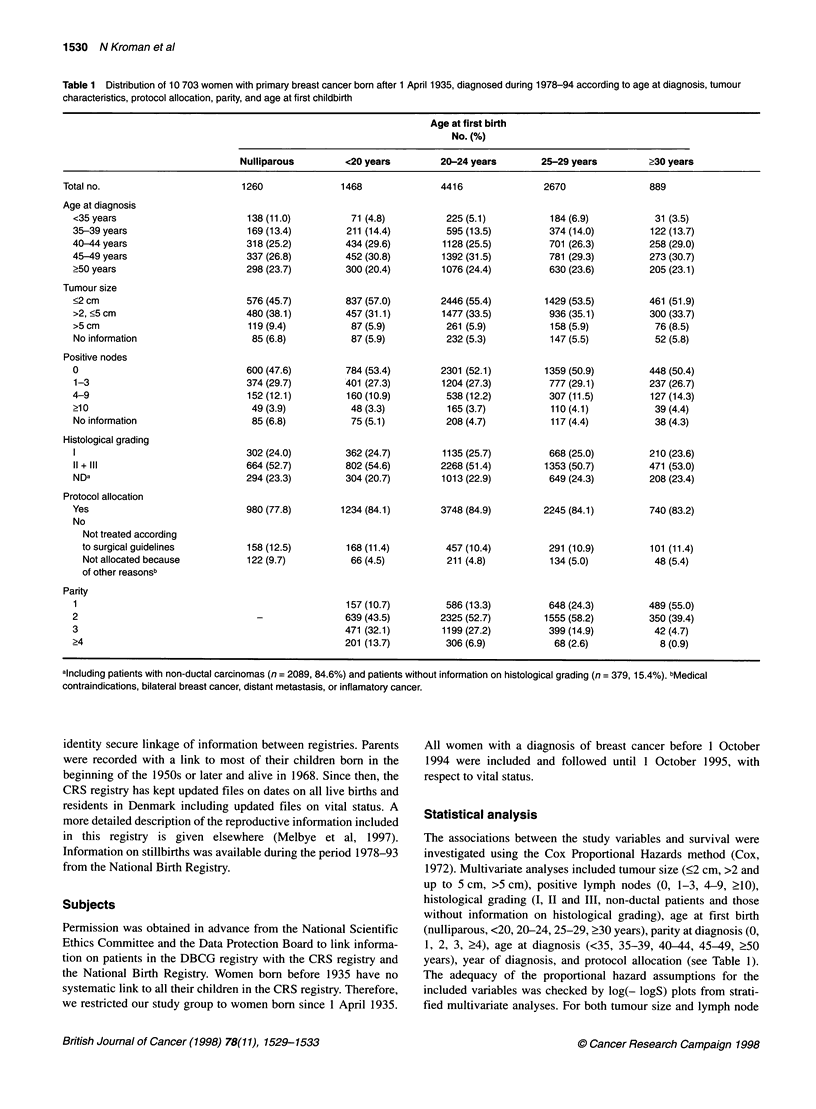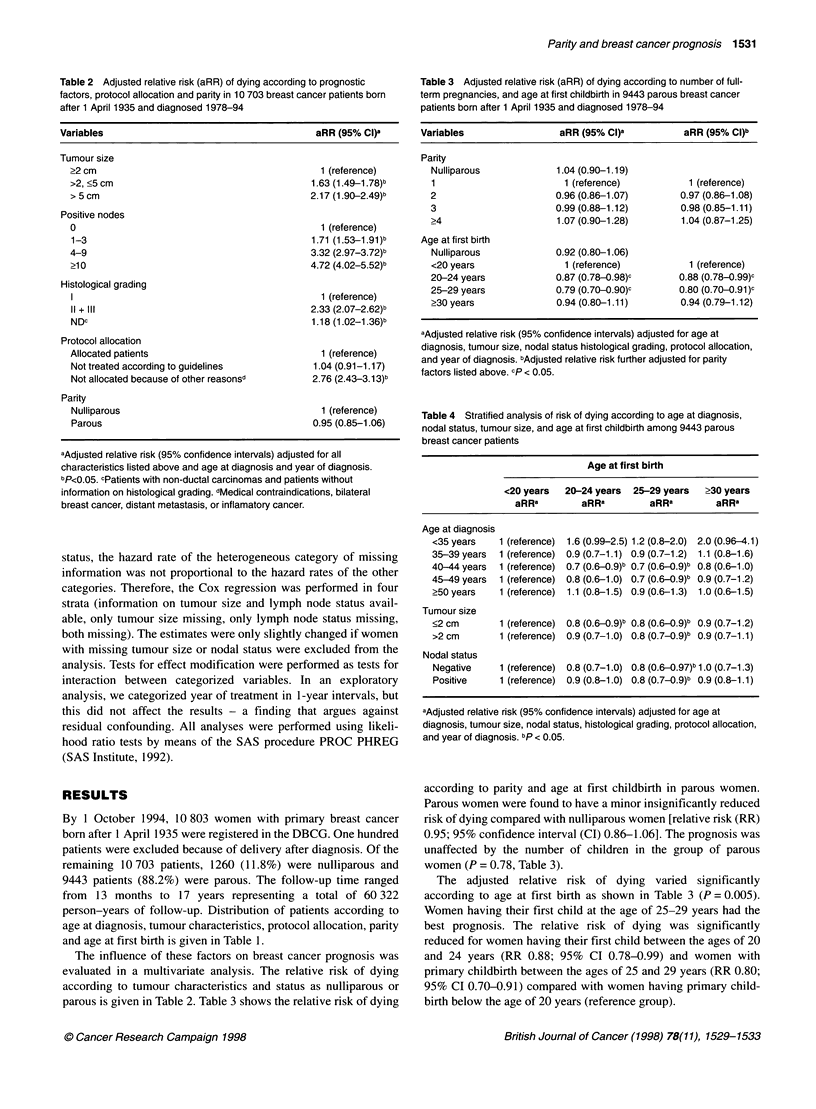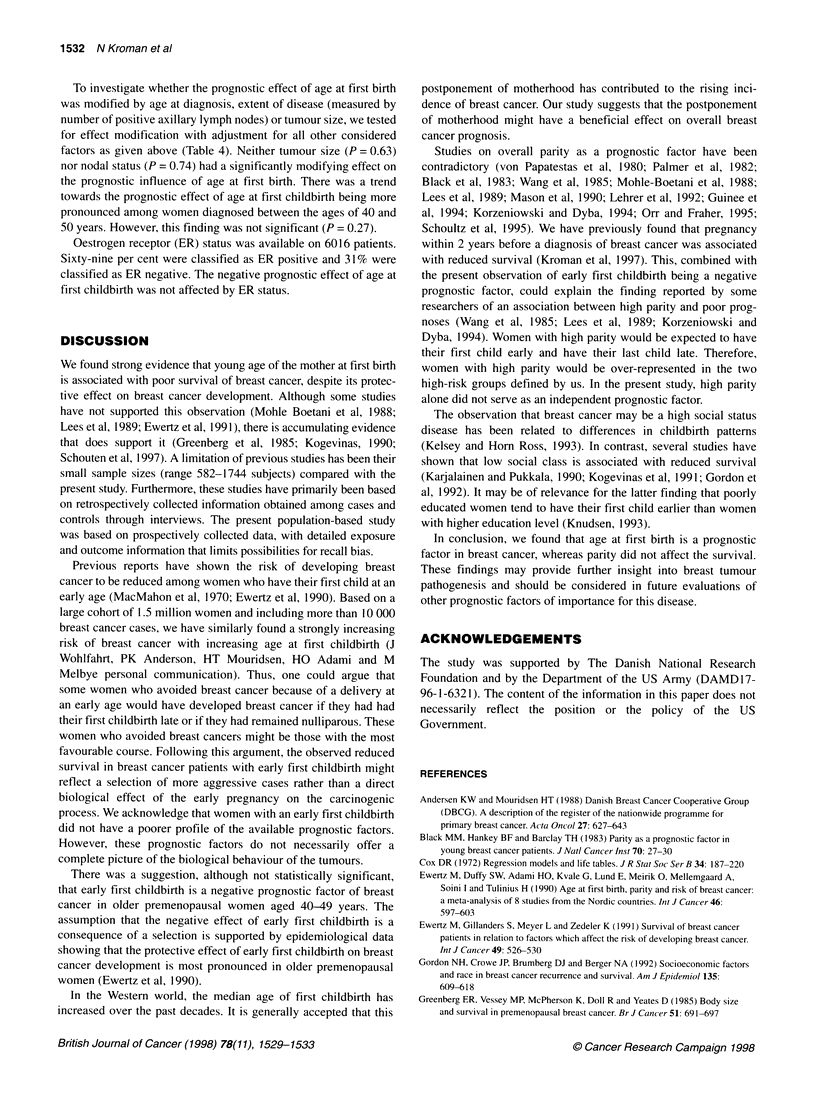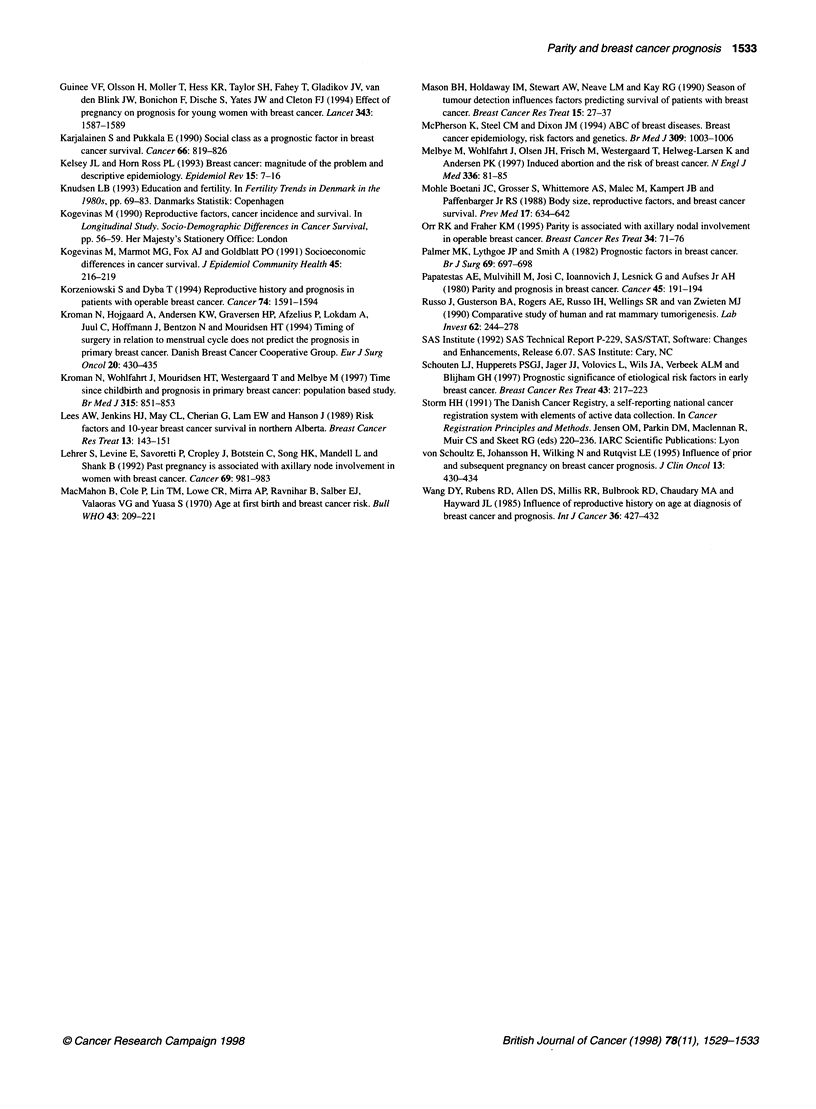# Parity, age at first childbirth and the prognosis of primary breast cancer.

**DOI:** 10.1038/bjc.1998.718

**Published:** 1998-12

**Authors:** N. Kroman, J. Wohlfahrt, K. W. Andersen, H. T. Mouridsen, T. Westergaard, M. Melbye

**Affiliations:** Department of Epidemiology Research, Danish Epidemiology Science Centre, Statens Serum Institut, Copenhagen.

## Abstract

Reproductive factors are known to be aetiologically important in breast cancer, but less is known regarding their effect on breast cancer prognosis. We have investigated the prognostic effect of age at first birth and total parity using data from the Danish Breast Cancer Cooperative Group that, since 1977, has collected population-based information on tumour characteristics, treatment regimes and follow-up status on Danish women with breast cancer. Details of pregnancy history were added from the Danish Civil Registration System and the National Birth Registry. Included in the study were 10,703 women with primary breast cancer. After adjusting for age and stage of disease (tumour size, axillary nodal status and histological grading), the number of full-term pregnancies was found without prognostic value. However, women with primary childbirth between 20 and 29 years experienced a significantly reduced risk of death compared with women with primary childbirth below the age of 20 years [20-24 years: relative risk (RR) = 0.88, 95% confidence interval (CI) 0.78-0.99; 25-29 years: RR = 0.80, 95% CI 0.70-0.91]. Further adjustment for oestrogen receptor status did not influence these results. The effect was not modified by age at diagnosis, tumour size or nodal status. In conclusion, low age at first childbirth, but not parity, was associated with a poor prognosis of breast cancer. We speculate whether women who develop breast cancer despite an early first full-term pregnancy might represent a selected group with a more malignant disease.


British Journal of Cancer (1998) 78(11), 1529–1533
© 1998 Cancer Research Campaign

### Parity, age at first childbirth and the prognosis of primary breast cancer

**N Kroman[1], J Wohlfahrt[1], K West Andersen[2], HT Mouridsen[2], T Westergaard[1] and M Melbye[1]**

[1]Department of Epidemiology Research, Danish Epidemiology Science Centre, Statens Serum Institut, Copenhagen, Denmark; [2]Danish Breast Cancer Cooperative Group, Rigshospitalet, Copenhagen, Denmark

**Summary** Reproductive factors are known to be aetiologically important in breast cancer, but less is known regarding their effect on breast cancer prognosis. We have investigated the prognostic effect of age at first birth and total parity using data from the Danish Breast Cancer Cooperative Group that, since 1977, has collected population-based information on tumour characteristics, treatment regimes and follow-up status on Danish women with breast cancer. Details of pregnancy history were added from the Danish Civil Registration System and the National Birth Registry. Included in the study were 10 703 women with primary breast cancer. After adjusting for age and stage of disease (tumour size, axillary nodal status and histological grading), the number of full-term pregnancies was found without prognostic value. However, women with primary childbirth between 20 and 29 years experienced a significantly reduced risk of death compared with women with primary childbirth below the age of 20 years [20–24 years: relative risk (RR) = 0.88, 95% confidence interval (CI) 0.78–0.99; 25–29 years: RR = 0.80, 95% CI 0.70–0.91]. Further adjustment for oestrogen receptor status did not influence these results. The effect was not modified by age at diagnosis, tumour size or nodal status. In conclusion, low age at first childbirth, but not parity, was associated with a poor prognosis of breast cancer. We speculate whether women who develop breast cancer despite an early first full-term pregnancy might represent a selected group with a more malignant disease.

**Keywords:** breast cancer; reproductive factors; survival; prognostic factors; oestrogen receptor

It is well-established that reproductive factors influence the risk of breast cancer development (McPherson et al, 1994). Based on animal studies, it has been hypothesized that pregnancy induces differentiation and maturation of the breast cells and that the cells subsequently become less vulnerable to carcinogenic stimuli (Russo et al, 1990). Parous women and in particular multiparous women are known to be at a lower risk of breast cancer than nulliparous women. Women having their first childbirth at a young age seem to experience a particular reduction in risk (MacMahon et al, 1970; Ewertz et al, 1990).

Factors influencing the development of breast cancer might also affect its course, but studies of the prognostic influence of reproductive factors have been contradictory (Papatestas et al, 1980; Palmer et al, 1982; Black et al, 1983; Wang et al, 1985; Mohle Boetani et al, 1988; Lees et al, 1989; Mason et al, 1990; Lehrer et al, 1992; Guinee et al, 1994; Korzeniowski and Dyba, 1994; Orr and Fraher, 1995; von Schoultz et al, 1995; Schouten et al, 1997). We took advantage of the population-based registration of breast cancer patients established by the Danish Breast Cancer Cooperative Group (DBCG) and a database containing complete information on parity to evaluate the possible importance of childbirth history and age at first birth as prognostic factors in primary breast cancer.

*Received 26 September 1997*
*Revised 28 December 1997*
*Accepted 7 January 1998*

*Correspondence to:* M Melbye, Department of Epidemiology Research, Danish Epidemiology Science Centre, Statens Serum Institut, Artillerivej 5, DK 2300 Copenhagen S, Denmark

### MATERIALS AND METHODS

### Population registries

Primary clinical and histopathological data together with data concerning post-operative therapy and follow-up have been registered by the DBCG since 1977 (Andersen and Mouridsen, 1988). The Danish Cancer Registry contains information on nearly all incident cases of malignant neoplasms diagnosed in Denmark since 1943 (Storm, 1991). The DBCG has information on 93% of all breast cancer patients born after 1 April 1935, reported to The Danish Cancer Registry.

The primary surgical treatment of the patients included total mastectomy plus axillary sampling (90% of cases), or lumpectomy with axillary sampling. Patients were hereafter classified as either low risk or high risk according to histopathological criteria. Treatment guidelines, strategy for risk group allocation and post-operative treatment have previously been described in detail (Andersen and Mouridsen, 1988; Kroman et al, 1994).

Patients with bilateral breast cancer, distant metastases, inflammatory cancer, with contraindication to the planned post-operative therapy, or patients who were not treated according to the surgical guidelines were not allocated to treatment protocols (miscellaneous group). The miscellaneous patient group could be separated into a group with a favourable prognosis and a group with a bad prognosis. Patients who were excluded from protocol allocation because of a surgical treatment that did not follow the guidelines had a better prognosis than patients excluded for other reasons.

Information on reproductive history was obtained by linkage with the Civil Registration System (CRS). The CRS was established on 1 April 1968 when all residents in Denmark were registered and assigned a unique identification number that permits

**Table 1** Distribution of 10 703 women with primary breast cancer born after 1 April 1935, diagnosed during 1978–94 according to age at diagnosis, tumour characteristics, protocol allocation, parity, and age at first childbirth

| | Age at first birth No. (%) | | | | |
|---|---|---|---|---|---|
| | **Nulliparous** | **<20 years** | **20–24 years** | **25–29 years** | **≥30 years** |
| Total no. | 1260 | 1468 | 4416 | 2670 | 889 |
| Age at diagnosis | | | | | |
| <35 years | 138 (11.0) | 71 (4.8) | 225 (5.1) | 184 (6.9) | 31 (3.5) |
| 35–39 years | 169 (13.4) | 211 (14.4) | 595 (13.5) | 374 (14.0) | 122 (13.7) |
| 40–44 years | 318 (25.2) | 434 (29.6) | 1128 (25.5) | 701 (26.3) | 258 (29.0) |
| 45–49 years | 337 (26.8) | 452 (30.8) | 1392 (31.5) | 781 (29.3) | 273 (30.7) |
| ≥50 years | 298 (23.7) | 300 (20.4) | 1076 (24.4) | 630 (23.6) | 205 (23.1) |
| Tumour size | | | | | |
| ≤2 cm | 576 (45.7) | 837 (57.0) | 2446 (55.4) | 1429 (53.5) | 461 (51.9) |
| >2, ≤5 cm | 480 (38.1) | 457 (31.1) | 1477 (33.5) | 936 (35.1) | 300 (33.7) |
| >5 cm | 119 (9.4) | 87 (5.9) | 261 (5.9) | 158 (5.9) | 76 (8.5) |
| No information | 85 (6.8) | 87 (5.9) | 232 (5.3) | 147 (5.5) | 52 (5.8) |
| Positive nodes | | | | | |
| 0 | 600 (47.6) | 784 (53.4) | 2301 (52.1) | 1359 (50.9) | 448 (50.4) |
| 1–3 | 374 (29.7) | 401 (27.3) | 1204 (27.3) | 777 (29.1) | 237 (26.7) |
| 4–9 | 152 (12.1) | 160 (10.9) | 538 (12.2) | 307 (11.5) | 127 (14.3) |
| ≥10 | 49 (3.9) | 48 (3.3) | 165 (3.7) | 110 (4.1) | 39 (4.4) |
| No information | 85 (6.8) | 75 (5.1) | 208 (4.7) | 117 (4.4) | 38 (4.3) |
| Histological grading | | | | | |
| I | 302 (24.0) | 362 (24.7) | 1135 (25.7) | 668 (25.0) | 210 (23.6) |
| II + III | 664 (52.7) | 802 (54.6) | 2268 (51.4) | 1353 (50.7) | 471 (53.0) |
| ND[a] | 294 (23.3) | 304 (20.7) | 1013 (22.9) | 649 (24.3) | 208 (23.4) |
| Protocol allocation | | | | | |
| Yes | 980 (77.8) | 1234 (84.1) | 3748 (84.9) | 2245 (84.1) | 740 (83.2) |
| No | | | | | |
| Not treated according to surgical guidelines | 158 (12.5) | 168 (11.4) | 457 (10.4) | 291 (10.9) | 101 (11.4) |
| Not allocated because of other reasons[b] | 122 (9.7) | 66 (4.5) | 211 (4.8) | 134 (5.0) | 48 (5.4) |
| Parity | | | | | |
| 1 | | 157 (10.7) | 586 (13.3) | 648 (24.3) | 489 (55.0) |
| 2 | – | 639 (43.5) | 2325 (52.7) | 1555 (58.2) | 350 (39.4) |
| 3 | | 471 (32.1) | 1199 (27.2) | 399 (14.9) | 42 (4.7) |
| ≥4 | | 201 (13.7) | 306 (6.9) | 68 (2.6) | 8 (0.9) |

[a]Including patients with non-ductal carcinomas ($n$ = 2089, 84.6%) and patients without information on histological grading ($n$ = 379, 15.4%). [b]Medical contraindications, bilateral breast cancer, distant metastasis, or inflamatory cancer.

identity secure linkage of information between registries. Parents were recorded with a link to most of their children born in the beginning of the 1950s or later and alive in 1968. Since then, the CRS registry has kept updated files on dates on all live births and residents in Denmark including updated files on vital status. A more detailed description of the reproductive information included in this registry is given elsewhere (Melbye et al, 1997). Information on stillbirths was available during the period 1978–93 from the National Birth Registry.

### Subjects

Permission was obtained in advance from the National Scientific Ethics Committee and the Data Protection Board to link information on patients in the DBCG registry with the CRS registry and the National Birth Registry. Women born before 1935 have no systematic link to all their children in the CRS registry. Therefore, we restricted our study group to women born since 1 April 1935. All women with a diagnosis of breast cancer before 1 October 1994 were included and followed until 1 October 1995, with respect to vital status.

### Statistical analysis

The associations between the study variables and survival were investigated using the Cox Proportional Hazards method (Cox, 1972). Multivariate analyses included tumour size (≤2 cm, >2 and up to 5 cm, >5 cm), positive lymph nodes (0, 1–3, 4–9, ≥10), histological grading (I, II and III, non-ductal patients and those without information on histological grading), age at first birth (nulliparous, <20, 20–24, 25–29, ≥30 years), parity at diagnosis (0, 1, 2, 3, ≥4), age at diagnosis (<35, 35–39, 40–44, 45–49, ≥50 years), year of diagnosis, and protocol allocation (see Table 1). The adequacy of the proportional hazard assumptions for the included variables was checked by log(– logS) plots from stratified multivariate analyses. For both tumour size and lymph node

 *© Cancer Research Campaign 1998*

**Table 2** Adjusted relative risk (aRR) of dying according to prognostic factors, protocol allocation and parity in 10 703 breast cancer patients born after 1 April 1935 and diagnosed 1978–94

| Variables | aRR (95% CI)[a] |
|---|---|
| Tumour size | |
| ≥2 cm | 1 (reference) |
| >2, ≤5 cm | 1.63 (1.49–1.78)[b] |
| > 5 cm | 2.17 (1.90–2.49)[b] |
| Positive nodes | |
| 0 | 1 (reference) |
| 1–3 | 1.71 (1.53–1.91)[b] |
| 4–9 | 3.32 (2.97–3.72)[b] |
| ≥10 | 4.72 (4.02–5.52)[b] |
| Histological grading | |
| I | 1 (reference) |
| II + III | 2.33 (2.07–2.62)[b] |
| ND[c] | 1.18 (1.02–1.36)[b] |
| Protocol allocation | |
| Allocated patients | 1 (reference) |
| Not treated according to guidelines | 1.04 (0.91–1.17) |
| Not allocated because of other reasons[d] | 2.76 (2.43–3.13)[b] |
| Parity | |
| Nulliparous | 1 (reference) |
| Parous | 0.95 (0.85–1.06) |

[a]Adjusted relative risk (95% confidence intervals) adjusted for all characteristics listed above and age at diagnosis and year of diagnosis. [b]$P<0.05$. [c]Patients with non-ductal carcinomas and patients without information on histological grading. [d]Medical contraindications, bilateral breast cancer, distant metastasis, or inflamatory cancer.

status, the hazard rate of the heterogeneous category of missing information was not proportional to the hazard rates of the other categories. Therefore, the Cox regression was performed in four strata (information on tumour size and lymph node status available, only tumour size missing, only lymph node status missing, both missing). The estimates were only slightly changed if women with missing tumour size or nodal status were excluded from the analysis. Tests for effect modification were performed as tests for interaction between categorized variables. In an exploratory analysis, we categorized year of treatment in 1-year intervals, but this did not affect the results – a finding that argues against residual confounding. All analyses were performed using likelihood ratio tests by means of the SAS procedure PROC PHREG (SAS Institute, 1992).

### RESULTS

By 1 October 1994, 10 803 women with primary breast cancer born after 1 April 1935 were registered in the DBCG. One hundred patients were excluded because of delivery after diagnosis. Of the remaining 10 703 patients, 1260 (11.8%) were nulliparous and 9443 patients (88.2%) were parous. The follow-up time ranged from 13 months to 17 years representing a total of 60 322 person–years of follow-up. Distribution of patients according to age at diagnosis, tumour characteristics, protocol allocation, parity and age at first birth is given in Table 1.

The influence of these factors on breast cancer prognosis was evaluated in a multivariate analysis. The relative risk of dying according to tumour characteristics and status as nulliparous or parous is given in Table 2. Table 3 shows the relative risk of dying according to parity and age at first childbirth in parous women. Parous women were found to have a minor insignificantly reduced risk of dying compared with nulliparous women [relative risk (RR) 0.95; 95% confidence interval (CI) 0.86–1.06]. The prognosis was unaffected by the number of children in the group of parous women ($P = 0.78$, Table 3).

The adjusted relative risk of dying varied significantly according to age at first birth as shown in Table 3 ($P = 0.005$). Women having their first child at the age of 25–29 years had the best prognosis. The relative risk of dying was significantly reduced for women having their first child between the ages of 20 and 24 years (RR 0.88; 95% CI 0.78–0.99) and women with primary childbirth between the ages of 25 and 29 years (RR 0.80; 95% CI 0.70–0.91) compared with women having primary childbirth below the age of 20 years (reference group).

**Table 3** Adjusted relative risk (aRR) of dying according to number of full-term pregnancies, and age at first childbirth in 9443 parous breast cancer patients born after 1 April 1935 and diagnosed 1978–94

| Variables | aRR (95% CI)[a] | aRR (95% CI)[b] |
|---|---|---|
| Parity | | |
| Nulliparous | 1.04 (0.90–1.19) | |
| 1 | 1 (reference) | 1 (reference) |
| 2 | 0.96 (0.86–1.07) | 0.97 (0.86–1.08) |
| 3 | 0.99 (0.88–1.12) | 0.98 (0.85–1.11) |
| ≥4 | 1.07 (0.90–1.28) | 1.04 (0.87–1.25) |
| Age at first birth | | |
| Nulliparous | 0.92 (0.80–1.06) | |
| <20 years | 1 (reference) | 1 (reference) |
| 20–24 years | 0.87 (0.78–0.98)[c] | 0.88 (0.78–0.99)[c] |
| 25–29 years | 0.79 (0.70–0.90)[c] | 0.80 (0.70–0.91)[c] |
| ≥30 years | 0.94 (0.80–1.11) | 0.94 (0.79–1.12) |

[a]Adjusted relative risk (95% confidence intervals) adjusted for age at diagnosis, tumour size, nodal status histological grading, protocol allocation, and year of diagnosis. [b]Adjusted relative risk further adjusted for parity factors listed above. [c]$P < 0.05$.

**Table 4** Stratified analysis of risk of dying according to age at diagnosis, nodal status, tumour size, and age at first childbirth among 9443 parous breast cancer patients

| | Age at first birth | | | |
|---|---|---|---|---|
| | <20 years aRR[a] | 20–24 years aRR[a] | 25–29 years aRR[a] | ≥30 years aRR[a] |
| Age at diagnosis | | | | |
| <35 years | 1 (reference) | 1.6 (0.99–2.5) | 1.2 (0.8–2.0) | 2.0 (0.96–4.1) |
| 35–39 years | 1 (reference) | 0.9 (0.7–1.1) | 0.9 (0.7–1.2) | 1.1 (0.8–1.6) |
| 40–44 years | 1 (reference) | 0.7 (0.6–0.9)[b] | 0.7 (0.6–0.9)[b] | 0.8 (0.6–1.0) |
| 45–49 years | 1 (reference) | 0.8 (0.6–1.0) | 0.7 (0.6–0.9)[b] | 0.9 (0.7–1.2) |
| ≥50 years | 1 (reference) | 1.1 (0.8–1.5) | 0.9 (0.6–1.3) | 1.0 (0.6–1.5) |
| Tumour size | | | | |
| ≤2 cm | 1 (reference) | 0.8 (0.6–0.9)[b] | 0.8 (0.6–0.9)[b] | 0.9 (0.7–1.2) |
| >2 cm | 1 (reference) | 0.9 (0.7–1.0) | 0.8 (0.7–0.9)[b] | 0.9 (0.7–1.1) |
| Nodal status | | | | |
| Negative | 1 (reference) | 0.8 (0.7–1.0) | 0.8 (0.6–0.97)[b] | 1.0 (0.7–1.3) |
| Positive | 1 (reference) | 0.9 (0.8–1.0) | 0.8 (0.7–0.9)[b] | 0.9 (0.8–1.1) |

[a]Adjusted relative risk (95% confidence intervals) adjusted for age at diagnosis, tumour size, nodal status, histological grading, protocol allocation, and year of diagnosis. [b]$P < 0.05$.

© Cancer Research Campaign 1998

To investigate whether the prognostic effect of age at first birth was modified by age at diagnosis, extent of disease (measured by number of positive axillary lymph nodes) or tumour size, we tested for effect modification with adjustment for all other considered factors as given above (Table 4). Neither tumour size ($P = 0.63$) nor nodal status ($P = 0.74$) had a significantly modifying effect on the prognostic influence of age at first birth. There was a trend towards the prognostic effect of age at first childbirth being more pronounced among women diagnosed between the ages of 40 and 50 years. However, this finding was not significant ($P = 0.27$).

Oestrogen receptor (ER) status was available on 6016 patients. Sixty-nine per cent were classified as ER positive and 31% were classified as ER negative. The negative prognostic effect of age at first childbirth was not affected by ER status.

### DISCUSSION

We found strong evidence that young age of the mother at first birth is associated with poor survival of breast cancer, despite its protective effect on breast cancer development. Although some studies have not supported this observation (Mohle Boetani et al, 1988; Lees et al, 1989; Ewertz et al, 1991), there is accumulating evidence that does support it (Greenberg et al, 1985; Kogevinas, 1990; Schouten et al, 1997). A limitation of previous studies has been their small sample sizes (range 582–1744 subjects) compared with the present study. Furthermore, these studies have primarily been based on retrospectively collected information obtained among cases and controls through interviews. The present population-based study was based on prospectively collected data, with detailed exposure and outcome information that limits possibilities for recall bias.

Previous reports have shown the risk of developing breast cancer to be reduced among women who have their first child at an early age (MacMahon et al, 1970; Ewertz et al, 1990). Based on a large cohort of 1.5 million women and including more than 10 000 breast cancer cases, we have similarly found a strongly increasing risk of breast cancer with increasing age at first childbirth (J Wohlfahrt, PK Anderson, HT Mouridsen, HO Adami and M Melbye personal communication). Thus, one could argue that some women who avoided breast cancer because of a delivery at an early age would have developed breast cancer if they had had their first childbirth late or if they had remained nulliparous. These women who avoided breast cancers might be those with the most favourable course. Following this argument, the observed reduced survival in breast cancer patients with early first childbirth might reflect a selection of more aggressive cases rather than a direct biological effect of the early pregnancy on the carcinogenic process. We acknowledge that women with an early first childbirth did not have a poorer profile of the available prognostic factors. However, these prognostic factors do not necessarily offer a complete picture of the biological behaviour of the tumours.

There was a suggestion, although not statistically significant, that early first childbirth is a negative prognostic factor of breast cancer in older premenopausal women aged 40–49 years. The assumption that the negative effect of early first childbirth is a consequence of a selection is supported by epidemiological data showing that the protective effect of early first childbirth on breast cancer development is most pronounced in older premenopausal women (Ewertz et al, 1990).

In the Western world, the median age of first childbirth has increased over the past decades. It is generally accepted that this postponement of motherhood has contributed to the rising incidence of breast cancer. Our study suggests that the postponement of motherhood might have a beneficial effect on overall breast cancer prognosis.

Studies on overall parity as a prognostic factor have been contradictory (von Papatestas et al, 1980; Palmer et al, 1982; Black et al, 1983; Wang et al, 1985; Mohle-Boetani et al, 1988; Lees et al, 1989; Mason et al, 1990; Lehrer et al, 1992; Guinee et al, 1994; Korzeniowski and Dyba, 1994; Orr and Fraher, 1995; Schoultz et al, 1995). We have previously found that pregnancy within 2 years before a diagnosis of breast cancer was associated with reduced survival (Kroman et al, 1997). This, combined with the present observation of early first childbirth being a negative prognostic factor, could explain the finding reported by some researchers of an association between high parity and poor prognoses (Wang et al, 1985; Lees et al, 1989; Korzeniowski and Dyba, 1994). Women with high parity would be expected to have their first child early and have their last child late. Therefore, women with high parity would be over-represented in the two high-risk groups defined by us. In the present study, high parity alone did not serve as an independent prognostic factor.

The observation that breast cancer may be a high social status disease has been related to differences in childbirth patterns (Kelsey and Horn Ross, 1993). In contrast, several studies have shown that low social class is associated with reduced survival (Karjalainen and Pukkala, 1990; Kogevinas et al, 1991; Gordon et al, 1992). It may be of relevance for the latter finding that poorly educated women tend to have their first child earlier than women with higher education level (Knudsen, 1993).

In conclusion, we found that age at first birth is a prognostic factor in breast cancer, whereas parity did not affect the survival. These findings may provide further insight into breast tumour pathogenesis and should be considered in future evaluations of other prognostic factors of importance for this disease.

### ACKNOWLEDGEMENTS

The study was supported by The Danish National Research Foundation and by the Department of the US Army (DAMD17-96-1-6321). The content of the information in this paper does not necessarily reflect the position or the policy of the US Government.

### REFERENCES

Andersen KW and Mouridsen HT (1988) Danish Breast Cancer Cooperative Group (DBCG). A description of the register of the nationwide programme for primary breast cancer. *Acta Oncol* **27**: 627–643

Black MM, Hankey BF and Barclay TH (1983) Parity as a prognostic factor in young breast cancer patients. *J Natl Cancer Inst* **70**: 27–30

Cox DR (1972) Regression models and life tables. *J R Stat Soc Ser B* **34**: 187–220

Ewertz M, Duffy SW, Adami HO, Kvale G, Lund E, Meirik O, Mellemgaard A, Soini I and Tulinius H (1990) Age at first birth, parity and risk of breast cancer: a meta-analysis of 8 studies from the Nordic countries. *Int J Cancer* **46**: 597–603

Ewertz M, Gillanders S, Meyer L and Zedeler K (1991) Survival of breast cancer patients in relation to factors which affect the risk of developing breast cancer. *Int J Cancer* **49**: 526–530

Gordon NH, Crowe JP, Brumberg DJ and Berger NA (1992) Socioeconomic factors and race in breast cancer recurrence and survival. *Am J Epidemiol* **135**: 609–618

Greenberg ER, Vessey MP, McPherson K, Doll R and Yeates D (1985) Body size and survival in premenopausal breast cancer. *Br J Cancer* **51**: 691–697

© Cancer Research Campaign 1998

Guinee VF, Olsson H, Moller T, Hess KR, Taylor SH, Fahey T, Gladikov JV, van den Blink JW, Bonichon F, Dische S, Yates JW and Cleton FJ (1994) Effect of pregnancy on prognosis for young women with breast cancer. *Lancet* **343**: 1587–1589

Karjalainen S and Pukkala E (1990) Social class as a prognostic factor in breast cancer survival. *Cancer* **66**: 819–826

Kelsey JL and Horn Ross PL (1993) Breast cancer: magnitude of the problem and descriptive epidemiology. *Epidemiol Rev* **15**: 7–16

Knudsen LB (1993) Education and fertility. In *Fertility Trends in Denmark in the 1980s*, pp. 69–83. Danmarks Statistik: Copenhagen

Kogevinas M (1990) Reproductive factors, cancer incidence and survival. In *Longitudinal Study. Socio-Demographic Differences in Cancer Survival*, pp. 56–59. Her Majesty's Stationery Office: London

Kogevinas M, Marmot MG, Fox AJ and Goldblatt PO (1991) Socioeconomic differences in cancer survival. *J Epidemiol Community Health* **45**: 216–219

Korzeniowski S and Dyba T (1994) Reproductive history and prognosis in patients with operable breast cancer. *Cancer* **74**: 1591–1594

Kroman N, Hojgaard A, Andersen KW, Graversen HP, Afzelius P, Lokdam A, Juul C, Hoffmann J, Bentzon N and Mouridsen HT (1994) Timing of surgery in relation to menstrual cycle does not predict the prognosis in primary breast cancer. Danish Breast Cancer Cooperative Group. *Eur J Surg Oncol* **20**: 430–435

Kroman N, Wohlfahrt J, Mouridsen HT, Westergaard T and Melbye M (1997) Time since childbirth and prognosis in primary breast cancer: population based study. *Br Med J* **315**: 851–853

Lees AW, Jenkins HJ, May CL, Cherian G, Lam EW and Hanson J (1989) Risk factors and 10-year breast cancer survival in northern Alberta. *Breast Cancer Res Treat* **13**: 143–151

Lehrer S, Levine E, Savoretti P, Cropley J, Botstein C, Song HK, Mandell L and Shank B (1992) Past pregnancy is associated with axillary node involvement in women with breast cancer. *Cancer* **69**: 981–983

MacMahon B, Cole P, Lin TM, Lowe CR, Mirra AP, Ravnihar B, Salber EJ, Valaoras VG and Yuasa S (1970) Age at first birth and breast cancer risk. *Bull WHO* **43**: 209–221

Mason BH, Holdaway IM, Stewart AW, Neave LM and Kay RG (1990) Season of tumour detection influences factors predicting survival of patients with breast cancer. *Breast Cancer Res Treat* **15**: 27–37

McPherson K, Steel CM and Dixon JM (1994) ABC of breast diseases. Breast cancer epidemiology, risk factors and genetics. *Br Med J* **309**: 1003–1006

Melbye M, Wohlfahrt J, Olsen JH, Frisch M, Westergaard T, Helweg-Larsen K and Andersen PK (1997) Induced abortion and the risk of breast cancer. *N Engl J Med* **336**: 81–85

Mohle Boetani JC, Grosser S, Whittemore AS, Malec M, Kampert JB and Paffenbarger Jr RS (1988) Body size, reproductive factors, and breast cancer survival. *Prev Med* **17**: 634–642

Orr RK and Fraher KM (1995) Parity is associated with axillary nodal involvement in operable breast cancer. *Breast Cancer Res Treat* **34**: 71–76

Palmer MK, Lythgoe JP and Smith A (1982) Prognostic factors in breast cancer. *Br J Surg* **69**: 697–698

Papatestas AE, Mulvihill M, Josi C, Ioannovich J, Lesnick G and Aufses Jr AH (1980) Parity and prognosis in breast cancer. *Cancer* **45**: 191–194

Russo J, Gusterson BA, Rogers AE, Russo IH, Wellings SR and van Zwieten MJ (1990) Comparative study of human and rat mammary tumorigenesis. *Lab Invest* **62**: 244–278

SAS Institute (1992) SAS Technical Report P-229, SAS/STAT, Software: Changes and Enhancements, Release 6.07. SAS Institute: Cary, NC

Schouten LJ, Hupperets PSGJ, Jager JJ, Volovics L, Wils JA, Verbeek ALM and Blijham GH (1997) Prognostic significance of etiological risk factors in early breast cancer. *Breast Cancer Res Treat* **43**: 217–223

Storm HH (1991) The Danish Cancer Registry, a self-reporting national cancer registration system with elements of active data collection. In *Cancer Registration Principles and Methods*. Jensen OM, Parkin DM, Maclennan R, Muir CS and Skeet RG (eds) 220–236. IARC Scientific Publications: Lyon von Schoultz E, Johansson H, Wilking N and Rutqvist LE (1995) Influence of prior and subsequent pregnancy on breast cancer prognosis. *J Clin Oncol* **13**: 430–434

Wang DY, Rubens RD, Allen DS, Millis RR, Bulbrook RD, Chaudary MA and Hayward JL (1985) Influence of reproductive history on age at diagnosis of breast cancer and prognosis. *Int J Cancer* **36**: 427–432

© Cancer Research Campaign 1998